# Loss of *Rb1* Enhances Glycolytic Metabolism in *Kras*-Driven Lung Tumors In Vivo

**DOI:** 10.3390/cancers12010237

**Published:** 2020-01-17

**Authors:** Lindsey R. Conroy, Susan Dougherty, Traci Kruer, Stephanie Metcalf, Pawel Lorkiewicz, Liqing He, Xinmin Yin, Xiang Zhang, Sengodagounder Arumugam, Lyndsay E.A. Young, Ramon C. Sun, Brian F. Clem

**Affiliations:** 1 Department of Biochemistry and Molecular Genetics, University of Louisville School of Medicine, Louisville, KY 40202, USA; 2 James Graham Brown Cancer Center, Louisville, KY 40202, USA; 3 Diabetes and Obesity Center, Christina Lee Brown Envirome Institute, Louisville, KY 40202, USA; 4 Department of Chemistry, University of Louisville, Center for Regulatory and Environmental Analytical Metabolomics, Louisville, KY 40208, USA; 5 Department of Molecular and Cellular Biochemistry, University of Kentucky, Lexington, KY 40536, USA; 6 Department of Neuroscience, University of Kentucky, College of Medicine, Lexington, KY 40536, USA; 7 Markey Cancer Center, Lexington, KY 40536, USA

**Keywords:** *Rb1*, metabolomics, glycolysis, TCA anaplerosis, lung cancer

## Abstract

Dysregulated metabolism is a hallmark of cancer cells and is driven in part by specific genetic alterations in various oncogenes or tumor suppressors. The retinoblastoma protein (pRb) is a tumor suppressor that canonically regulates cell cycle progression; however, recent studies have highlighted a functional role for pRb in controlling cellular metabolism. Here, we report that loss of the gene encoding pRb (*Rb1*) in a transgenic mutant *Kras*-driven model of lung cancer results in metabolic reprogramming. Our tracer studies using bolus dosing of [U-^13^C]-glucose revealed an increase in glucose carbon incorporation into select glycolytic intermediates. Consistent with this result, *Rb1*-depleted tumors exhibited increased expression of key glycolytic enzymes. Interestingly, loss of *Rb1* did not alter mitochondrial pyruvate oxidation compared to lung tumors with intact *Rb1*. Additional tracer studies using [U-^13^C,^15^N]-glutamine and [U-^13^C]-lactate demonstrated that loss of *Rb1* did not alter glutaminolysis or utilization of circulating lactate within the tricarboxylic acid cycle (TCA) in vivo. Taken together, these data suggest that the loss of *Rb1* promotes a glycolytic phenotype, while not altering pyruvate oxidative metabolism or glutamine anaplerosis in *Kras*-driven lung tumors.

## 1. Introduction

Lung cancer is the leading cause of cancer-related deaths for both men and women worldwide, with the 5-year survival rate being less than 18% [1]. Of the lung cancer subtypes, non-small-cell lung cancer (NSCLC) accounts for 85% of all lung cancer diagnoses [2]. Deregulation of the cell cycle is a major driver of tumorigenesis, and aberrant expression of cell cycle proteins in the retinoblastoma protein–E2F factor (pRb–E2F) pathway has been found to play a key role in the pathogenesis of NSCLC [3,4]. pRb is a tumor suppressor that is reported to be dysfunctional in the majority of human cancers. Canonically, pRb functions to regulate cell cycle progression by repressing the transcriptional activity of the E2F family of transcription factors, thereby inhibiting S-phase entry [5]. In most NSCLC cases, pRb inactivation occurs via hyperphosphorylation; however, in 15–30% of cases, *RB1* is mutated, which correlates to poor overall survival for these patients [6,7].

Advances in our understanding of pRb function have highlighted additional biochemical pathways under pRb regulation beyond cell cycle progression. Emerging evidence supports a direct role for pRb in regulating metabolic pathways, such as glycolysis, glutaminolysis, lipogenesis, mitochondrial oxidative phosphorylation, and reactive oxygen species metabolism [8,9,10,11]. pRb can exert its metabolic function by interacting with the E2F family of transcription factors and altering the expression of metabolic enzymes and transporters [12,13,14]. Additionally, acute loss of *Rb1* increases mitochondrial pyruvate oxidation in normal lung tissue; however, the metabolic effects of *Rb1* loss during lung cancer development are largely unknown.

Herein, we report that loss of *Rb1* in a mutant *Kras*-driven model of lung cancer enhances glycolytic metabolism without altering mitochondrial pyruvate oxidation. Moreover, loss of *Rb1* has no significant effect on TCA anaplerosis or utilization of alternative nutrient sources apart from glucose. These data expand our knowledge of understanding of the metabolic phenotype resulting from pRb dysfunction in a widely used model of lung cancer.

## 2. Results

### 2.1. Steady-State Metabolomics Highlights Metabolic Discrepancies in Rb1-Deficient Lung Tumors In Vivo

**Figure 1:**
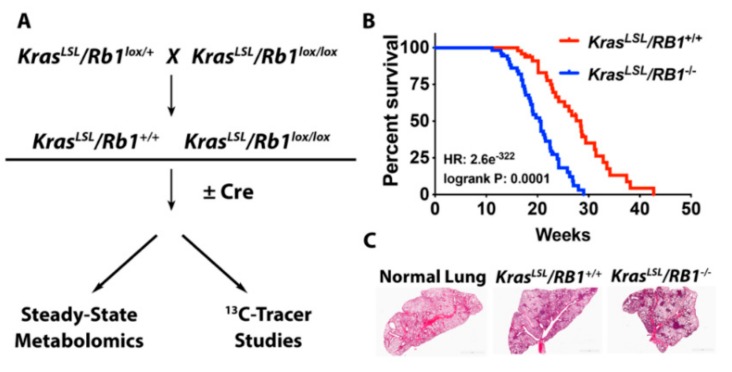
Loss of *Rb1* accelerates lung cancer progression in a *Kras*-driven model of lung cancer. (**A**) Schematic of experimental design to assess the metabolic function of pRb in *Kras*-driven lung tumors in vivo. For all experiments, non-instilled *Kras^LSL^/Rb1^lox/lox^* mice served as normal lung controls. (**B**) Kaplan–Meier survival analysis for *Rb1* wild-type (*Rb1^+/+^*) (*n* = 53) or knock-out (*Rb1^−/−^*) (*n* = 47) *Kras^LSL^* mice. (**C**) Representative H&E staining of lung tissue from normal, *Rb1^+/+^*, and *Rb1^−/−^* mice (*n* = 3).

The loss of *Rb1* accelerates lung tumor progression in mutant *Kras*-driven lung cancer in vivo, resulting in the development of higher grade adenocarcinomas and decreased overall survival [15]. Based on the emerging evidence that pRb directly regulates metabolism, we hypothesized that the loss of *Rb1* promotes a metabolic phenotype that supports tumor progression. We have utilized a combination of steady-state and stable isotope-labeled metabolomics to assess global changes in metabolism resulting from pRb dysfunction in *Kras*-driven lung tumors in vivo (Figure 1A).

**Figure 2:**
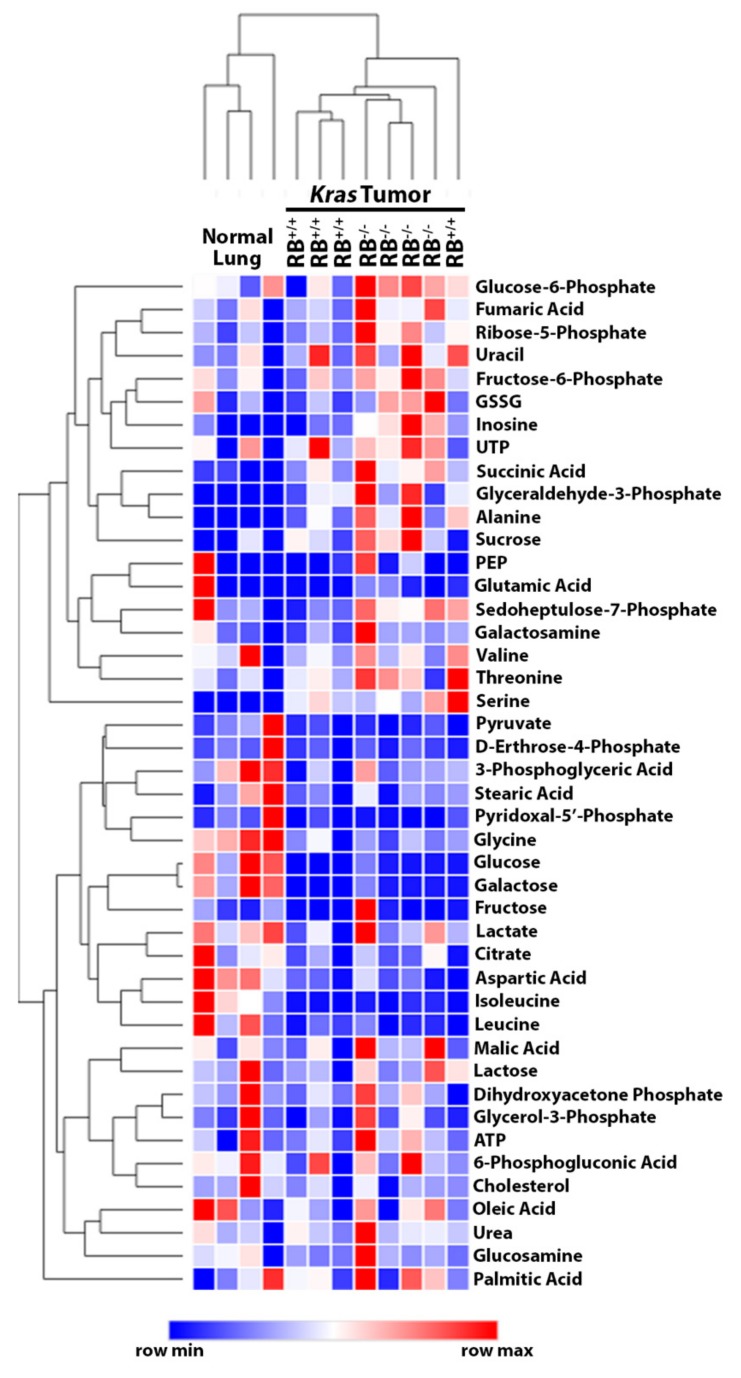
*Rb1* loss qualitatively alters the steady state relative abundance of metabolites in *Kras*-driven lung tumors. Hierarchical clustering analysis depicts the relative abundance of metabolites in normal lung, *Rb1^+/+^*, and *Rb1^−/−^* lung tumors (*n* = 4). Color coding indicates the row minimum or maximum for each metabolite from least (blue) to most (red) abundant.

Consistent with previous findings [15], loss of *Rb1* significantly decreased overall survival and increased tumor burden in this *Kras*-driven lung cancer model (Figure 1B,C; Supplementary Figure S1). To identify potential pRb-dependent metabolic adaptations in these lung tumors, we performed a steady-state metabolomics analysis to analyze the relative abundance of metabolites within major metabolic pathways. Hierarchical clustering demonstrated distinct metabolic patterns between normal lung, *Rb1^+/+^*, and *Rb1^−/−^* lung tumors (Figure 2). Interestingly, glucose-6-phosphate, fructose-6-phsophate, glyceraldehyde-3-phosphate, and metabolites within the pentose phosphate pathway (ribose-5-phosphate) appeared to be elevated in the *Rb1^−/−^* lung tumors. This suggested that pRb may regulate glucose utilization in *Kras*-driven lung tumors as many of the qualitative changes in metabolite abundance were observed within glycolysis or metabolic pathways originating from glycolytic intermediates.

### 2.2. Loss of Rb1 Enhances Glycolysis in Kras-Driven Lung Tumors

**Figure 3:**
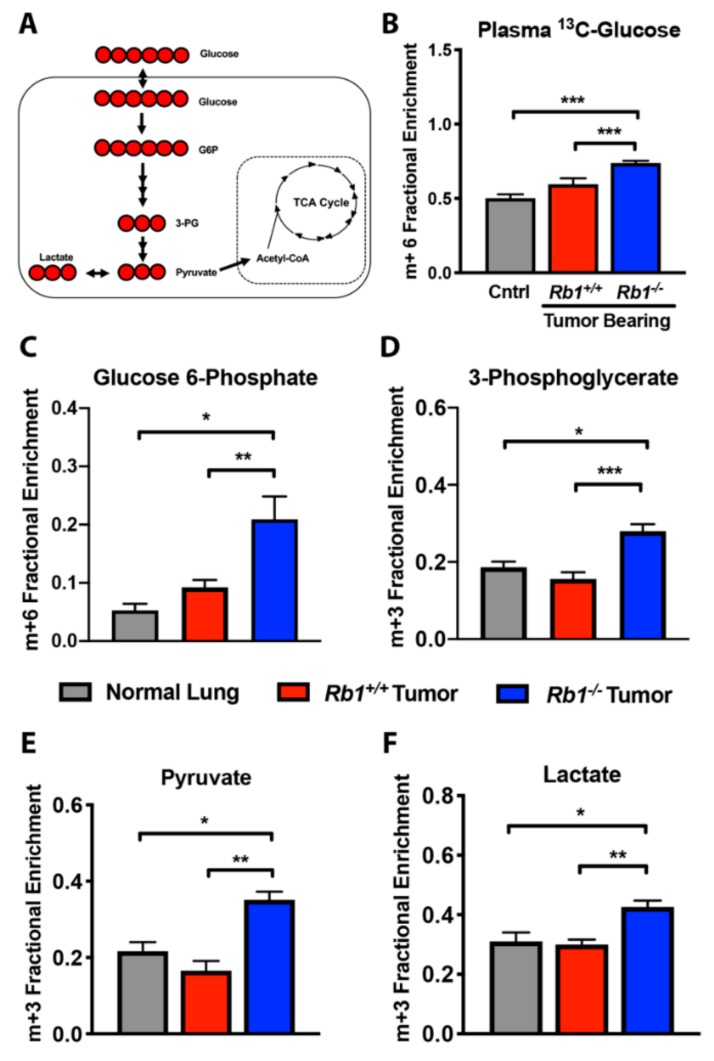
Loss of *Rb1* increases glucose carbon incorporation into glycolytic intermediates in vivo. (**A**) Cartoon of [U-^13^C]-glucose fate mapping through glycolysis. Red circles are ^13^C. (**B**) Fractional enrichment of fully labeled glucose (m+6) in plasma from control, *Rb1^+/+^*, and *Rb1*^−*/*−^ mice. (**C**–**F**) Fractional enrichment of m+6-labeled glucose 6-phosphate (**C**) and m+3-labeled 3-phosphoglycerate (**D**), pyruvate (**E**), and lactate (**F**) in normal lung, *Rb1^+/+^*, and *Rb1*^−*/*−^ lung tumors following bolus [U-^13^C]-glucose injections. For (**B**–**F**), values represent mean ± SEM analyzed by one-way ANOVA with Tukey’s post-hoc comparison (normal lung, *n* = 3; *Rb1^+/+^*, *n* = 12; *Rb1*^−/−^, *n* = 10). Statistical significances between each group are as follows: * *p* < 0.05, ** *p* < 0.01, or *** *p* < 0.001.

To examine differences in glucose utilization between *Rb1^+/+^* and *Rb1^−/−^* lung tumors, we preformed [U-^13^C]-glucose tracer studies. [U-^13^C]-glucose plasma enrichment was observed for both normal lung and tumor-bearing mice (Figure 3B). The utilization of ubiquitously labeled glucose results in the intracellular generation of ^13^C labeling of hexose and triose sugar intermediates within the glycolytic pathway, resulting in fully labeled pyruvate (m+3). Pyruvate can then be metabolized to lactate by lactate dehydrogenase (LDH), transaminated to alanine by alanine aminotransferase (ALT), or enter the TCA cycle through pyruvate dehydrogenase (PDH) or pyruvate carboxylase (PC). It has been previously shown that *Kras*-driven lung tumors display similar labeling of pyruvate and lactate as adjacent lung tissue [16]. Consistent with these findings, labeling of glycolytic intermediates from glucose carbon did not significantly differ in *Rb1^+/+^* tumors compared to normal lung tissue (Figure 3A,C–F). Conversely, loss of *Rb1* significantly increased glucose carbon incorporation into several glycolytic intermediates, including both pyruvate and lactate (Figure 3E,F).

**Figure 4:**
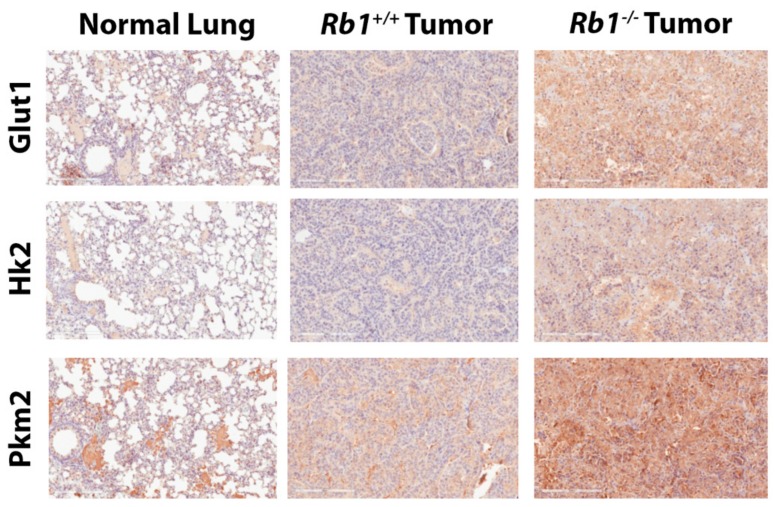
Loss of *Rb1* increases the expression of key glycolytic enzymes in *Kras*-driven lung tumors in vivo. Immunohistochemical staining to examine glucose transporter 1 (Glut1), hexokinase 2 (Hk2), and pyruvate kinase M2 (Pkm2) expression in normal lung, *Rb1^+/+^*, and *Rb1*^−*/*−^ lung tumors. Images were taken under 20X magnification and the scale bar in lower left corner of each image represents 200 μm. Staining is representative of *n* = 3.

We next sought to determine if the observed increase in carbon labeling of glycolytic intermediates in *Rb1*^−*/*−^ lung tumors was due to changes in the expression of rate-limiting enzymes in *Kras*-driven lung tumors. We performed an immunohistochemistry analysis for glucose transporter 1 (Glut1), hexokinase 2 (Hk2), and pyruvate kinase M2 (Pkm2) in both normal and lung tumor tissues. We found that loss of *Rb1* qualitatively increased Glut1, Hk2, and Pkm2 in *Kras*-driven lung tumors compared to those with intact *Rb1* and normal lung tissue (Figure 4). These results indicated that *Rb1* deletion enhanced glycolysis, in part, via upregulation of glycolytic enzymes in *Kras*-driven lung tumors in vivo.

**Figure 5:**
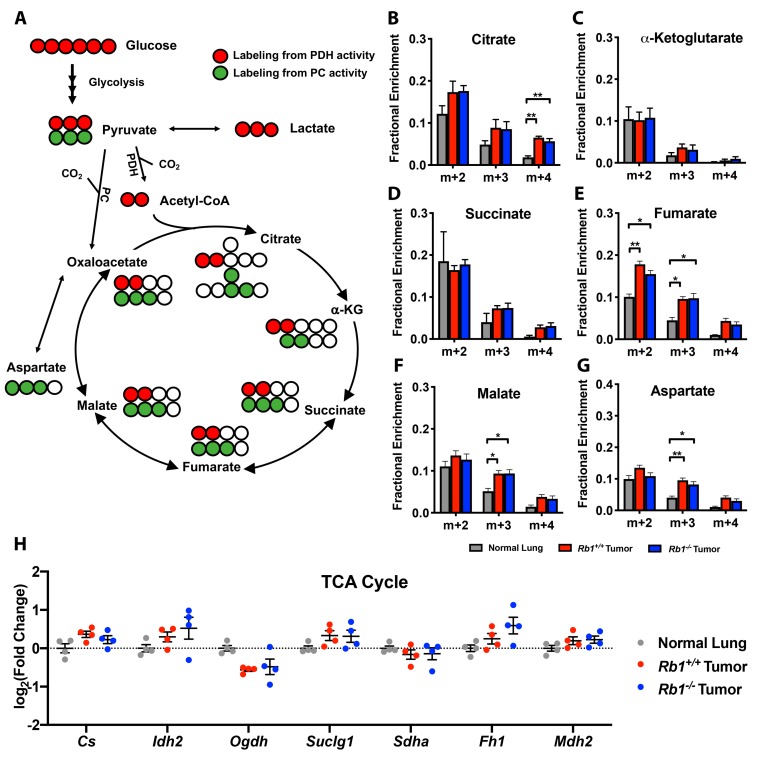
Loss of *Rb1* does not affect pyruvate metabolism in *Kras*-driven lung tumors in vivo. (**A**) Cartoon of [U-^13^C]-glucose fate mapping through the TCA cycle. Red circles are ^13^C labeling indicative of pyruvate dehydrogenase activity, green circles are ^13^C labeling indicative of pyruvate carboxylase activity, and white circles are unlabeled ^12^C. (**B**–**G**) Fractional enrichment of TCA metabolites in normal lung, *Rb1^+/+^*, and *Rb1*^−*/*−^ lung tumors (normal lung, *n* = 3; *Rb1^+/+^*, *n* = 12; *Rb1*^−*/*−^, *n* = 10). (**H**) qPCR analysis of the TCA cycle genes citrate synthase (*Cs*), isocitrate dehydrogenase 2 (*Idh2*), alpha-ketoglutarate dehydrogenase (*Ogdh*), succinate-CoA ligase alpha subunit (*Suclg1*), succinate dehydrogenase complex subunit A (*Sdha*), fumarate hydratase 1 (*Fh1*), and malate dehydrogenase 2 (*Mdh2*) in normal lung, *Rb1^+/+^*, and *Rb1*^−*/*−^ lung tumors (*n* = 4). For (**B**–**H**), values represent mean ± SEM analyzed by one-way ANOVA with Tukey’s post-hoc comparison. Statistical significances between each group are as follows: * *p* < 0.05, ** *p* < 0.01.

As stated earlier, pyruvate carbon can enter the TCA cycle via two distinct mechanisms. Pyruvate can enter the TCA cycle as acetyl-CoA generated from the pyruvate dehydrogenase complex (PDH), or via anaplerosis in which pyruvate enters the TCA cycle as oxaloacetate through the activity of pyruvate carboxylase (PC) (Figure 5A). PDH entry of pyruvate carbon is indicated by m+2 (1st turn)/m+4 (2nd turn) isotopologues, while PC activity is observed by m+3 isotopologue labeling of TCA intermediates. *Kras*-driven lung tumors utilize pyruvate as the primary source of TCA cycle carbon via PDH and exhibit elevated PC activity [16,17]. Consistent with these studies, we observed increased m+2 and m+4 carbon labeling in certain intermediates as well as m+3 aspartate, malate, and fumarate in *Rb1^+/+^* lung tumors compared to normal lung tissue (Figure 5E–G). As several studies have demonstrated a role for the pRb–E2F pathway in regulating oxidative metabolism [12,13,14,18], we sought to define if loss of *Rb1* further contributes to pyruvate oxidation in *Kras*-driven lung tumors. Interestingly, we observed no significant difference in both PDH- or PC-mediated pyruvate carbon entry into the TCA cycle between *Rb1^+/+^* and *Rb1*^−*/*−^ lung tumors in vivo (Figure 5B–G). Moreover, loss of *Rb1* did not alter the gene expression of TCA cycle enzymes (Figure 5H). Taken together, the analysis of [U-^13^C]-glucose labeling in pRb-deficient lung tumors suggested that the loss of *Rb1* enhanced glycolysis without altering mitochondrial pyruvate oxidation in vivo.

### 2.3. Rb1 Does Not Regulate Glutamine Utilization In Vivo

**Figure 6:**
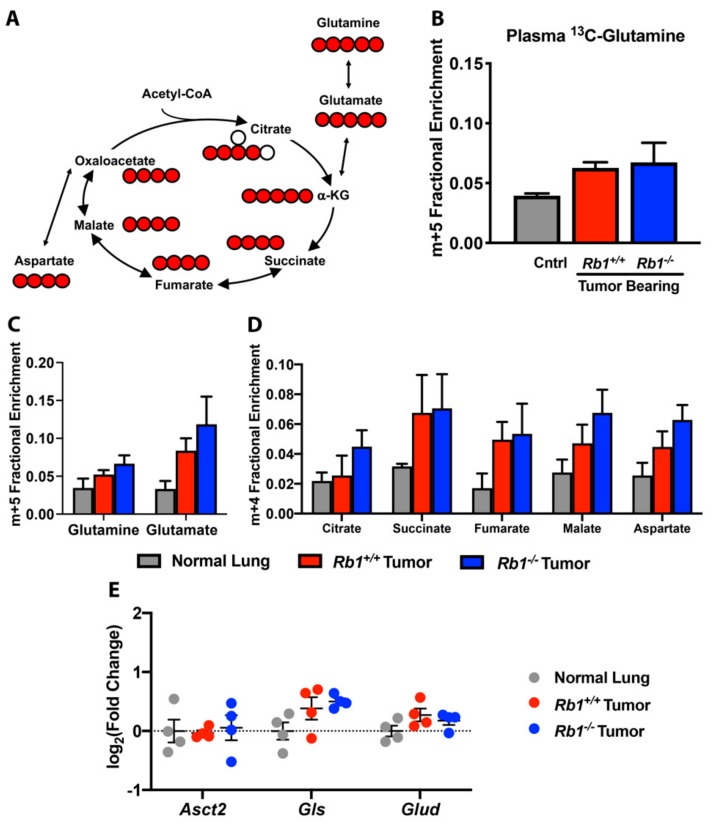
Loss of *Rb1* does not influence glutaminolysis in vivo. (**A**) Cartoon of [U-^13^C,^15^N]-glutamine fate mapping through the TCA cycle. Red circles are ^13^C and white circles are unlabeled ^12^C. (**B**) Fractional enrichment of fully labeled glutamine (m+5) in plasma from control, *Rb1^+/+^*, and *Rb1^−/−^* mice. (**C**) Fractional enrichment of fully labeled glutamine (m+5) and glutamate (m+5) in normal lung, *Rb1^+/+^*, and *Rb1^−/−^* lung tumors following bolus [U-^13^C,^15^N]-glutamine injections. (**D**) Fractional enrichment of m+4 TCA metabolites in normal lung, *Rb1^+/+^* and *Rb1^–/–^* lung tumors. (**E**) qPCR analysis of glutamine transporter (*Asct2*), glutaminase (*Gls*), and glutamate dehydrogenase (*Glud1*) in normal lung, *Rb1^+/+^*, and *Rb1^–/–^* tumors. For (**B**–**E**), values represent mean ± SEM analyzed by one-way ANOVA with Tukey’s post-hoc comparison (*n* = 4).

Although it has been demonstrated that *Kras*-driven lung tumors do not utilize glutamine in vivo [16], loss of *Rb1* has been shown to increase glutamine utilization as a source of anaplerotic carbon for the TCA cycle in mouse embryonic fibroblasts (MEFs) [19]. As such, we sought to examine differences in glutamine utilization between *Rb1^+/+^* and *Rb1*^−*/*−^ lung tumors using [U-^13^C,^15^N]-glutamine tracer studies. Under three sequential bolus doses, we observed low plasma [U-^13^C,^15^N]-glutamine and intracellular labeled glutamine and glutamate for both normal lung and tumor-bearing mice (Figure 6A–C). As has been previously reported for *Kras*-driven lung tumors [16], we found no significant increase in glutamine anaplerosis in TCA cycle intermediates. Interestingly, pRb-deficient tumors did not exhibit significantly different labeling of TCA cycle intermediates compared to *Kras*-driven lung tumors or normal lung tissue (Figure 6A,D). Consistent with this finding, we did not observe any significant expression changes in genes encoding glutamine transporter (*Asct2*), glutaminase (*Gls*), or glutamate dehydrogenase (*Glud1*) (Figure 6E). Together, our findings suggested that pRb did not influence glutamine utilization in the context of *Kras*-driven lung cancer in vivo.

### 2.4. Loss of Rb1 Does Not Alter Lactate Utilization in Kras-Driven Lung Tumors

**Figure 7:**
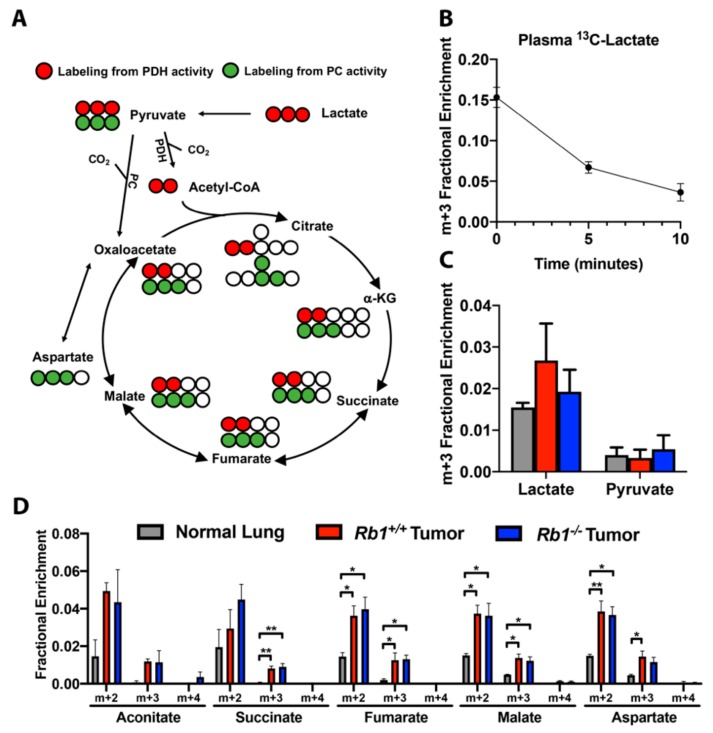
**p**Rb does not affect lactate carbon oxidation in *Kras*-driven lung tumors in vivo. (**A**) Cartoon of [U-^13^C]-lactate fate mapping through the TCA cycle. Red circles are ^13^C labeling indicative of pyruvate dehydrogenase activity, green circles are ^13^C labeling indicative of pyruvate carboxylase activity, and white circle are unlabeled ^12^C. (**B**) Fractional enrichment of fully labeled lactate (m+3) in plasma from control mice following bolus [U-^13^C]-lactate injections. Mice were euthanized immediately (0 min), 5, or 10 min after the last injection (*n* = 2 for each time point). (**C**) Fractional enrichment of fully labeled lactate (m+3) and pyruvate (m+3) in normal lung, *Rb1^+/+^*, and *Rb1^–/–^* lung tumors following bolus [U-^13^C]-lactate injections (*n* = 4). (**D**) Fractional enrichment of TCA metabolites in normal lung, *Rb1^+/+^* and *Rb1^–/–^* lung tumors (*n* = 4). For (**B**–**D**), values represent mean ± SEM analyzed by one-way ANOVA with Tukey’s post-hoc comparison. Statistical significances between each group are as follows: * *p* < 0.05 or ** *p* < 0.01.

Recent studies have reported that circulating lactate can be utilized as a TCA cycle carbon source in both *Kras*-driven lung tumors in vivo as well as in NSCLC patients [20,21]. Given that the loss of *Rb1* does not alter mitochondrial pyruvate oxidation in animals administered labeled glucose (Figure 5), we hypothesized that the loss of *Rb1* may influence the utilization of circulating lactate. For these studies, we developed a labeling protocol using [U-^13^C]-lactate based on the method used for our [U-^13^C]-glucose and [U-^13^C,^15^N]-glutamine tracer studies. In brief, three bolus doses of [U-^13^C]-lactate were administered via tail vein injection, each 15 min apart. Fifteen minutes after the last injection, mice were euthanized and lung tumors or normal lung tissue was harvested for metabolite extraction followed by 2D-LC-MS/MS analysis. Using this approach, plasma ^13^C-lactate was undetectable in both normal lung and tumor-bearing mice when euthanized 15 min after the last injection. This is likely in part due to the high turnover rate for circulating lactate [21]. To confirm that [U-^13^C]-lactate was being rapidly turned over, we euthanized animals at 0, 5, or 10 min after the third dose of [U-^13^C]-lactate. When euthanized immediately, plasma ^13^C-lactate reached approximately 15%, then rapidly decreased by 5 and 10 min (Figure 7B). Even under these conditions, we were able to observe intracellular labeling of lactate-derived metabolites (Figure 7C,D). Although tumor-bearing and normal lung mice exhibited similar levels of m+3 isotopologues of lactate and pyruvate (Figure 7A,C), we observed increased m+2 and m+3 isotopologue labeling of several TCA cycle intermediates consistent with *Kras*-driven lung tumors exhibiting increased lactate utilization as a nutrient source for the TCA cycle (Figure 7A,D). The loss of *Rb1* did not significantly alter the extent of metabolite labeling, suggesting that pRb did not contribute to the control of lactate metabolism within the mitochondria of *Kras*-driven lung tumors in vivo.

## 3. Discussion

In the current study, we report that the loss of *Rb1* in mutant *Kras*-driven lung tumors leads to a metabolic shift in which pRb-deficient tumors display enhanced glycolysis presumably via upregulation of the glucose transporter, Glut1 and two rate-limiting enzymes in glycolysis, Hk2 and Pkm2. Interestingly, this increase in labeled glycolytic intermediates in *Rb1*^−*/*−^ tumors compared to *Rb1^+/+^* tumors was not observed in downstream metabolites within the TCA cycle. This was unexpected, as studies have previously shown that *Rb1* deletion results in increased TCA cycle activity and mitochondrial activation in non-cancer tissues in vivo [14,22]. Conversely, other studies have reported that the pRb/E2F1 axis directly controls PDH activity by modulating the expression of pyruvate dehydrogenase kinase 4, suggesting that the loss of pRb function should decrease glucose flux into the TCA cycle [12,13]. Although we observed a significant increase in m+3 pyruvate labeling from glucose in pRb-deficient tumors, we found no accompanying increase in the labeling of TCA intermediates between *Rb1*^−*/*−^ and *Rb1^+/+^* tumors (Figure 3E and Figure 5B–G). A similar increase in labeled lactate was also observed. Additional tracer studies to examine lactate utilization also yielded no significant difference in labeling of TCA cycle intermediates in pRb-deficient tumors compared to those with intact pRb. Beyond pRb, many oncogenes and tumor suppressors have been reported to directly influence cellular metabolism. *KRAS* is mutated in a large portion of NSCLC and its activation results in metabolic rewiring, including increased oxidative phosphorylation. While pRb has been previously described to control mitochondrial glucose oxidation [13,14], this may be obscured by the activity of mutant K-ras which may explain the lack of additional labeling in *Kras*-driven tumors upon loss of *Rb1*. The depletion of pRb can still lead to elevated expression of glycolytic enzymes in this context and increased glycolytic activity. Defining how combinatorial actions of different oncogenic or tumor suppressors converge in regulating tumor metabolism would greatly impact our understanding of tumor biology.

It has recently been suggested that the decoupling of mitochondrial oxidative phosphorylation from glycolysis provides a selective advantage to cancer cells to sustain cell proliferation [20,21]. The enhanced conversion of pyruvate to lactate regenerates the necessary oxidized nicotinamide adenine dinucleotide (NAD+) required for elevated glycolysis to persist, while the use of circulating lactate for mitochondrial respiration operates in parallel to support the energetic functions of the TCA cycle [20]. Our studies support the use of lactate as an oxidative fuel within the mitochondria of *Kras*-driven tumors, as has been previously reported [20]. We found substantial labeling in circulating lactate in the plasma of animals administered [U-^13^C]-glucose (m+3; 40–45%). We further observed similar labeling patterns of TCA intermediates from mice administered either bolus [U-^13^C]-glucose or [U-^13^C]-lactate (Figure 5B–G and Figure 7D), wherein loss of *Rb1* had no significant effect. The disparity in overall labeling enrichment in TCA intermediates between either glucose or lactate administration is presumed to be due to differences in the levels of circulating labeled lactate produced under these bolus dosing conditions. The use of circulating lactate as a fuel for the TCA cycle may allow glucose to supply other anabolic pathways for cell growth. Specifically, glucose serves as a carbon source for the synthesis of nucleotides, amino acids, and lipids for biomass accumulation [23,24]. pRb has been reported to regulate nucleotide metabolism via its interaction with E2F family members [8]. The observed increase in aerobic glycolysis in pRb-deficient tumors could be to sustain nucleotide synthesis; however, the labeling duration used in our tracer studies was not sufficient to see the incorporation of glucose carbon into nucleotides, therefore additional studies will need to be performed to assess the role of pRB in regulating nucleotide metabolism in vivo. Overall, our data indicated that loss of *Rb1* enhances glycolysis, but does not influence pyruvate oxidation in the context of *Kras*-driven lung tumors in vivo. It would be of interest to expand these findings into NSCLC patient samples. There have been previous reports of metabolic changes in human NSCLC following labeled glucose administration, but no reports of genotypic analysis beyond the presence of mutant *KRAS*. We would anticipate that defining the potential pRb status in these samples would aid in correlating human findings to our results from these animal studies. Yet, this will be challenging given the heterogeneous nature of human NSCLC compared to these defined genetic mouse models, but could potentially be a focus for future studies.

While aerobic glycolysis is a known feature of cancer cells, targeting this pathway for therapy has proven challenging due to the cytotoxicity of many anti-glycolytic agents [25]. Biguanides, such as metformin and phenformin, are common therapeutics for the treatment of type 2 diabetes, and have recently been reported to exhibit anticancer activity in vitro and in vivo [26,27,28]. Specifically, both agents have an inhibitory effect on glycolysis and oxidative metabolism. The addition of metformin to standard therapy for lung adenocarcinoma patients significantly improves patient outcome [29], while phenformin inhibits tumor growth and angiogenesis in preclinical models of *Kras*-driven NSCLC [30]. As it extends to these results, our data suggested that patients with mutant K-ras/pRb-deficient lung tumors may further benefit from combination therapy that are inclusive of either metformin or phenformin treatment. This strategy can be incorporated into future studies in defining the therapeutic response to metabolic disruptors in these mouse models in order to support the translational potential of these findings.

The role of pRb in immunity has been well described [31,32], with recent studies demonstrating that pRb inactivation recruits tumor-associated macrophages and immunosuppressive myeloid-derived suppressor cells within the tumor microenvironment (TME) [33]. This was found to be due to increased cytokine/chemokine secretion through altered AMP-activated protein kinase signaling from increased fatty acid oxidation. These studies strongly suggest a tumor-derived metabolic contribution in regulating the immune landscape. Beyond the effects on cell proliferation, tumor cell-derived lactate produced during aerobic glycolysis has a profound effect on the tumor microenvironment and cancer progression [34]. Extracellular lactate has deleterious effects on infiltrating T-cells, while serving as a nutrient source for immunosuppressive tumor-associated macrophages [34,35,36]. pRb-deficient tumors exhibited increased lactate production from glucose (Figure 3F), yet whether this influences the immune response in the TME remains unclear.

The loss of *Rb1* in vitro promotes glutamine utilization via upregulation of the glutamine transporter Asct2 and glutaminase in MEFs [19]. This phenomenon was not observed in vivo. pRb-deficient *Kras*-driven tumors neither exhibited significant differences in glutamine carbon labeling of TCA cycle intermediates compared to lung tumors with intact pRb nor increased the expression of genes essential for glutamine utilization (Figure 6D). It has been reported that, while glutamine strongly contributes to oxidative metabolism in cultured cells, circulating glutamine is not utilized as a primary fuel source for TCA anaplerosis in vivo, particularly in *Kras*-driven lung tumors [16]. This may explain, in part, the discrepancy in glutamine utilization between the MEFs and transgenic lung tumor model and suggest that the metabolic functions of pRb may be dependent on the in vivo environment of tumor cells.

Our studies demonstrated a role for pRb in regulating glycolytic metabolism in *Kras*-driven lung tumors in vivo. Using both steady-state metabolomics and stable-isotope tracer studies, we report that the loss of *Rb1* enhances glycolysis without altering mitochondrial pyruvate oxidation. Additionally, our studies demonstrated that pRb does not regulate glutamine anaplerosis in vivo. Together, our data highlighted a metabolic phenotype resulting from pRb dysfunction in *Kras*-driven lung tumors and may support the targeting of glycolytic metabolism in NSCLC patients with pRb-deficient tumors.

## 4. Materials and Methods

### 4.1. Mouse Model and Adenoviral Infection

All animal studies were approved by the University of Louisville’s Institutional Animal Care and Use Committee (protocol 16674). *Kras^LSL/G12D^*/*Rb1^+/+^* and *Kras^LSL/G12D^*/*Rb1^lox/lox^* mice were generated by breeding *Kras^LSL/G12D^*/*Rb1^+/lox^* mice as described in [15]. Lung tumors were induced in 8-week-old *Kras^LSL/G12D^*/*Rb1^+/+^* and *Kras^LSL/G12D^*/*Rb1^lox/lox^* mice by intratracheal instillation of 2.5 × 10^7^ PFU of an adenovirus expressing Cre recombinase (Vector Development Lab, Baylor College of Medicine, Houston, TX, USA). For all studies, age-matched, non-instilled *Kras^LSL/G12D^*/*Rb1^lox/lox^* mice were used as normal lung controls.

### 4.2. Kaplan-Meier Analysis

Eight-weeks post-surgery, *Kras^LSL/G12D^*/*Rb1^+/+^* and *Kras^LSL/G12D^*/*Rb1^lox/lox^* mice were examined twice weekly. Mice were sacrificed upon visible signs of advanced morbidity, such as lethargy, weight loss (>15%), hunching, and distressed rapid breathing. The log-rank *p*-value and hazard ratio (HR) were calculated using Kaplan–Meier survival analysis in GraphPad Prism software (Version 8.3.0., San Diego, CA, USA).

### 4.3. [U-^13^C]-Glucose, [U-^13^C,^15^N]-Glutamine, and [U-^13^C]-Lactate Tracer Studies

Upon tumor onset (*Kras^LSL/G12D^*/*Rb1^lox/lox^*: 10–16 weeks; *Kras^LSL/G12D^*/*Rb1^+/+^*: 15–20 weeks; determined by symptomatic observation of labored breathing), mice were injected via the tail vein with either 80 µL of 25% *w/v* [U-^13^C]-glucose or 200 µL of 36.2 mg/mL [U-^13^C,^15^N]-glutamine, three times, 15 min apart, as described in [37,38]. [U-^13^C]-lactate studies were performed following the same protocol using 100 µL of 300 mM [U-^13^C]-lactate. At 15 min after the last injection, mice were sacrificed by cervical dislocation, blood was collected, and lung tumors or normal lung were excised and flash frozen in liquid nitrogen. The number of animals per group (normal lung, *Rb1^+/+^* and *Rb1^–/–^* lung tumors) for each labeled nutrient is depicted within their respective figure legends.

### 4.4. Plasma [U-^13^C]-Glucose, [U-^13^C,^15^N]-Glutamine, and [U-^13^C]-Lactate Analysis

Blood was collected from mice by cardiac puncture following euthanasia by cervical dislocation and processed as described in [38]. Up to 150 µL of blood was collected in K_2_-EDTA microtubes, incubated at room temperature for 5 min, and then placed on ice. Plasma was separated from blood cells by centrifugation at 3500× *g* for 15 min at 4 °C. For [U-^13^C]-glucose and [U-^13^C]-lactate studies, plasma was deproteinized by trichloroacetic acid (TCA) extraction by adding 300 µL of 1:10 TCA dilution to 30 µL of plasma. Samples were centrifuged at 15,000 rpm for 30 min at 4 °C and the supernatant was vacuum dried by lyophilization. Samples were re-dissolved in 650 µL of D_2_O and analyzed by 2D-NMR. For [U-^13^C,^15^N]-glutamine studies, metabolites were extracted from plasma by adding 130 µL of methanol:water (80:20) to 10 µL plasma. Samples were vortexed for 10 s and incubated for 10 min at 4 °C, followed by centrifugation at 16,000× *g* for 10 min at 4 °C. The supernatant was vacuum dried by SpeedVac, followed by 2D-LC-MS/MS analysis.

### 4.5. Metabolite Tissue Sample Preparation for Tracer Studies

To extract metabolites from tissue samples, up to 20 mg of pulverized frozen tissue was extracted for polar and lipid metabolites as described in [37] with minor modifications. In brief, metabolites were extracted in acetonitrile:water:chloroform (1 mL:750 µL:500 µL). Samples were centrifuged at 3000× *g* for 20 min at 4 °C to separate the polar, lipid, and tissue debris layers. The remaining tissue debris was re-extracted with 500 µL of chloroform:methanol:butylated hydroxytoluene (2:1:1 mM) and centrifuged at 22,000× *g* for 20 min at 4 °C. The residual polar and lipid fractions were combined with their respective fractions from the first extraction. The polar fraction was vacuum-dried by lyophilization. The dried sample was dissolved in 100 µL of 50% acetonitrile and vigorously vortex-mixed for 3 min. After centrifugation at 14,000 rpm and 4 °C for 20 min, 80 µL of supernatant was collected for 2D-LC-MS/MS analysis.

### 4.6. Sample Preparation and Derivatization for Steady-State Metabolomics

Up to 20 mg of pulverized frozen tissue was extracted in 1 mL of 50% methanol and separated into a polar fraction (aqueous layer) and a protein/DNA/RNA/glycogen pellet. The polar fraction was dried at 10^–3^ mBar using SpeedVac (Thermo, Waltham, MA, USA) followed by derivatization. The protein/DNA/RNA/glycogen pellet was washed 4 times with 50% methanol and once with 100% methanol. Samples were centrifuged at 15,000 rpm for 10 min between washes. The hydrolysis of the protein/DNA/RNA/glycogen pellet was performed by first resuspending the dried pellet in deionized H_2_O followed by the addition of equal parts of 2N HCl. Samples were vortexed thoroughly and incubated at 95 °C for 2 h. The reaction was quenched with 100% methanol with 40 μM L-norvaline (as an internal control). The sample was incubated on ice for 30 min and the supernatant was collected by centrifugation at 15,000 rpm at 4 °C for 10 min. The collected supernatant was subsequently dried by a vacuum centrifuge at 10^−3^ mBar.

Dried polar and hydrolyzed pellet samples were derivatized by the addition of 50 μL of 20 mg/mL of methoxyamine in pyridine to the dried sample in a 1.5 mL Eppendorf tube. Samples were incubated for 1 h and 30 min at 30 °C. Tubes were then centrifuged at 15,000 rpm for 10 min. The supernatant of each tube was transferred to a v-shaped amber glass chromatography vial. Lastly, the addition of 80 μL of N-methyl-N-(trimethylsilyl)trifluoroacetamide (MSTFA) occurred with an incubation period at 37 °C for 30 min. The derivatized samples were then analyzed by GC-MS.

### 4.7. GC-MS Analysis and Data Processing

GC-MS protocols were similar to those described previously [39,40], except a modified temperature gradient was used for GC: Initial temperature was 130 °C, held for 4 min, rising at 6 °C/min to 243 °C, rising at 60 °C/min to 280 °C, held for 2 min. The electron ionization (EI) energy was set to 70 eV. Scan (*m*/*z*: 50–800) and full scan mode were used for the metabolomics analysis. Mass spectra were translated to relative metabolite abundance using the automated mass spectral deconvolution and identification system (AMDIS, developed by the National Institute of Standards and Technology, Gaithersburg, MD, USA) software matched to the FiehnLib metabolomics library (available through Agilent, Santa Clara, CA, USA) for retention time and fragmentation pattern matching with a confidence score greater than 80 [41,42,43]. Data were further analyzed using the data extraction for stable isotope-labeled metabolites (DEXSI) software package [44]. Relative abundance was corrected for recovery using L-norvaline and adjusted to protein input. The rapid quantitation of derivatized protein/DNA/RNA/glycogen pellet was performed as described in [45]. An unsupervised hierarchical clustering analysis of steady-state metabolite levels was performed using the online Morpheus software tool from the Broad Institute.

### 4.8. 2D-LC-MS/MS Analysis and Data Processing

All samples were randomly analyzed on a Thermo Q Exactive HF Hybrid Quadrupole-Orbitrap Mass Spectrometer coupled with a Thermo DIONEX UltiMate 3000 HPLC system (Thermo Fisher Scientific, Waltham, MA, USA). The UltiMate 3000 HPLC system was equipped with a reversed-phase chromatography (RPC) column and a hydrophilic interaction chromatography (HILIC) column that were configured in parallel to form a parallel 2D-LC-MS system [46]. To obtain full MS data, every sample was analyzed by parallel 2D-LC-MS in positive mode (+) and negative mode (−). For metabolite identification, one unlabeled sample in each sample group was analyzed by 2D-LC-MS/MS in positive mode (+) and negative mode (−) to acquire MS/MS spectra at three collision energies (20, 40, and 60 eV).

### 4.9. Data Analysis for 2D-LC-MS/MS

Full MS. raw files were first converted to mzML format with msConvert tool, a part of an open-source ProteoWizard suite, described in detail by Adusumilli and Mallick [47]. Isotopologue peak deconvolution and assignments were performed using El-MAVEN [48]. Peaks were assigned using a metabolite list generated and verified using full-scan MS and MS/MS spectra of unlabeled samples, as described previously [49,50,51]. The metabolite list contained metabolite names and corresponding molecular formulae used to generate theoretical *m*/*z* values for all possible isotopologues, and retention times for each metabolite. El-MAVEN parameters for compound library matching were as follows—Extracted-Ion Chromatogram (EIC) Extraction Window: ±7 ppm; Match Retention Time: ±0.60 min. For ^13^C isotopologue peak detection, the software criteria were set as follows—Minimum Isotope-Parent Correlation: 0.20, Isotope is within 5 scans of parent, Abundance Threshold: 1.0, and Maximum % Error to Natural Abundance: 100%. All assignments were visually inspected and compared to unlabeled samples for reference. The peak list with corresponding abundances was exported to a comma-separated (CSV) file and uploaded to the Polly workflow for natural abundance correction and calculation of total pool size for each metabolite (by summing peak areas of each detected isotopologue) using the Polly IsoCorrect module (developed by Elucidata, Cambridge, MA, USA). Finally, the data were downloaded and plotted using Microsoft Excel and GraphPad Prism software (Version 8.3.0., San Diego, CA, USA).

### 4.10. 2D-NMR Analysis

NMR spectra were recorded at 293 K at 14.1 T initially on a Varian Inova NMR spectrometer (Agilent, Santa Clara, CA, USA) with a 5 mm HCN cold probe and later on a Bruker Advance Neo NMR spectrometer equipped with a 5 mm Prodigy probe (Bruker, Billerica, MA, USA). Typically, pre-saturation at very low power was employed in a pulse sequence during the recycle delay (3 s) to saturate any residual water (HOD, arising due to exchange of protons with D_2_O and/or presence of trace amounts of H_2_O) in the samples before acquiring for about 2 s. Generally, 256 scans were performed on each sample for signal averaging for better signal-to-noise ratio. Labeled and unlabeled lactate and glucose concentrations were measured by simple integration of the peak areas of these metabolites relative to the known concentration of DSS (sodium trimethylsilylpropanesulfonate as a sodium salt) that is added to each sample, which serves as both chemical shift reference and metabolite quantification standard.

### 4.11. Real Time-PCR

Total RNA was isolated from up to 30 mg of frozen pulverized tissue using the RNeasy Mini Kit (Qiagen, Hilden, Germany) according to the manufacturer’s protocol. The resulting total RNA (1 μg) was converted to cDNA using the High-Capacity RNA-to-cDNA Kit (Applied Biosystems, Foster City, CA, USA). Gene expression was determined by qPCR using the following Taqman Gene Expression Assays: *Cs* (Mm00466043_m1), *Idh2* (Mm00612429_m1), *Ogdh* (Mm01179923_m1), *Suclg1* (Mm00451244_m1), *Sdha* (Mm01352366_m1), *Fh1* (Mm01321349_m1), *Mdh2* (Mm00725890_s1), *Asct2* (Mm00436603_m1), *Gls* (Mm01257297_m1), *Glud1* (Mm00492353_m1), and *β-actin* (Mm00607939_s1). *β-actin* was used as an internal control. Data are reported as the log (base 2) of the fold change.

### 4.12. Immunohistochemistry

Mice were sacrificed by cervical dislocation and lungs were harvested, fixed overnight in 4% paraformaldehyde, and paraffin-embedded. Lung tissue sections were dewaxed and rehydrated, followed by antigen retrieval in Tris-EDTA buffer (pH 9.0). Sections were then blocked using 5% goat serum and incubated with 1:100 dilution of the following antibodies overnight at 4 °C: Glut1 (ProteinTech, Rosemont, IL, USA, Cat. No. 21829-1-AP) Hk2 (Cell Signaling, Danvers, MA, USA, Cat. No. 2867S), and Pkm2 (Cell Signaling, Cat. No. 4053S). Sections were subsequently incubated with 1:500 dilution of an horseradish peroxidase (HRP)-conjugated anti-rabbit antibody (Invitrogen, Cat. No. 32260) for one hour at room temperature. Expression was detected using the 3,3′-diaminobenzidine (DAB) stain (Vector Laboratories, Berlingame, CA, USA) and counterstained with hematoxylin. Hematoxylin and eosin staining was performed by the University of Louisville’s Special Procedures Laboratory within the Department of Pathology. Imaging was performed using an Aperioscope digital slide scanner (Leica Biosystems, Buffalo Grove, IL, USA).

### 4.13. Immunoblotting

Protein lysate was generated from three distinct lung tumors from both *Kras/Rb^+/+^* and *Kras/Rb^–/–^* mice. Twenty micrograms of tumor or MCF-7 (positive control) protein lysate was separated by 10% SDS-PAGE followed by transfer to a polyvinylidene fluoride (PVDF) membrane. After transfer, membranes were cut between the 85 kDa and 64 kDa molecular weights for simultaneous assessment of Rb1, p107, and GAPDH using anti-RB (Cell Signaling, #9309, 1:1000), p107 (ProteinTech, 13354-1-AP, 1:500), and GAPDH (Millipore, Burlington, MA, USA, ABS16, 1:500) antibodies, respectively. Proteins were visualized using 1:5000 dilution of either anti-mouse or anti-rabbit HRP-linked secondary antibody and ECL Prime chemiluminescent reagent (GE Healthcare, Chicago, IL, USA).

### 4.14. Statistical Analysis

Statistical analyses were carried out using GraphPad Prism (Version 8.3.0., San Diego, CA, USA). All numerical data are reported as mean ± SEM. Grouped analysis was performed using one-way ANOVA with Tukey’s post-hoc comparison. For each experiment, replicates and p-values for all results are listed in their respective figure legends.

## 5. Conclusions

Our data demonstrates that loss of *Rb1* in *Kras*-driven lung tumors enhances glycolytic metabolism via upregulation of key glycolytic genes. Additionally, *Rb1* deletion does not have an effect on TCA cycle activity or differential nutrient utilization in vivo. Taken together, our studies have identified potential metabolic vulnerabilities resulting from pRb loss in lung cancer.

## Supplementary Materials

The following are available online at https://www.mdpi.com/2072-6694/12/1/237/s1.
