# Supplementary material for: Loss of Rb1 Enhances Glycolytic Metabolism in Kras-Driven Lung Tumors In Vivo
Source: Cancers (Basel). 2020 Jan 17;12(1):237. doi: 10.3390/cancers12010237 (PMC7016860; doi:10.3390/cancers12010237)
Supplement: Supplementary file 1 [file cancers-12-00237-s001.pdf]

Supplementary Materials: Loss of *Rb1* Enhances Glycolytic Metabolism in *Kras*-Driven Lung Tumors in Vivo

Lindsey R. Conroy, Susan Dougherty, Traci Kruer, Stephanie Metcalf, Pawel Lorkiewicz, Liqing He, Xinmin Yin, Xiang Zhang, Sengodagounder Arumugam, Lyndsay E.A. Young, Ramon C. Sun and Brian F. Clem

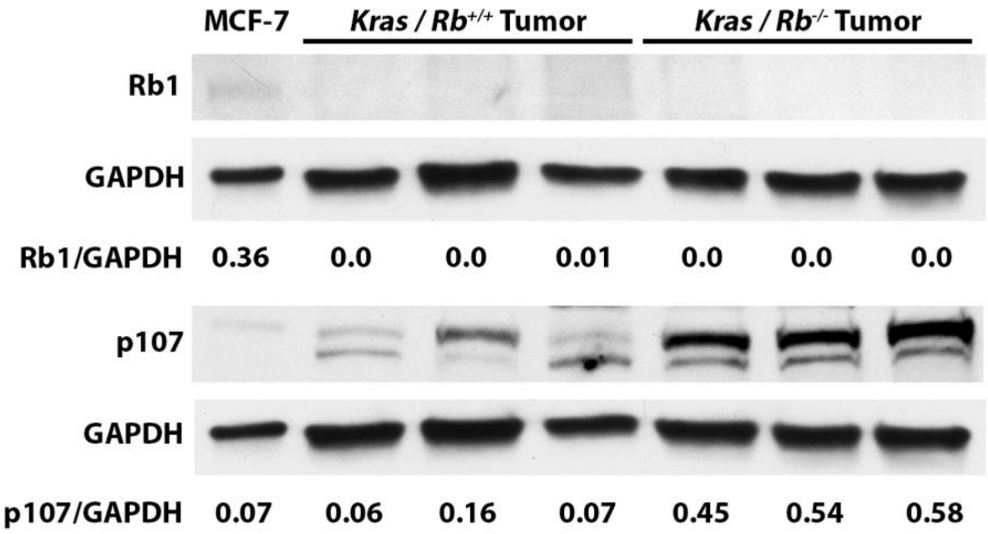

Figure S1. Confirmation of loss of *Rb1* in resulting *Kras*-driven lung tumors. Western blot analysis of Rb1 and p107 in three separate lung tumors after intratracheal instillation of Ad-Cre in either the *Kras*/*Rb*$^{+/+}$ or *Kras*/*Rb*$^{-/-}$ mice. While Rb1 expression is relatively low in all developing lung tumors, elevated p107 confirms loss of *Rb1* in the *Kras*/*Rb*$^{-/-}$ tumors as has been previously demonstrated for this model [1]. MCF-7 cell lysate was used as a positive control.

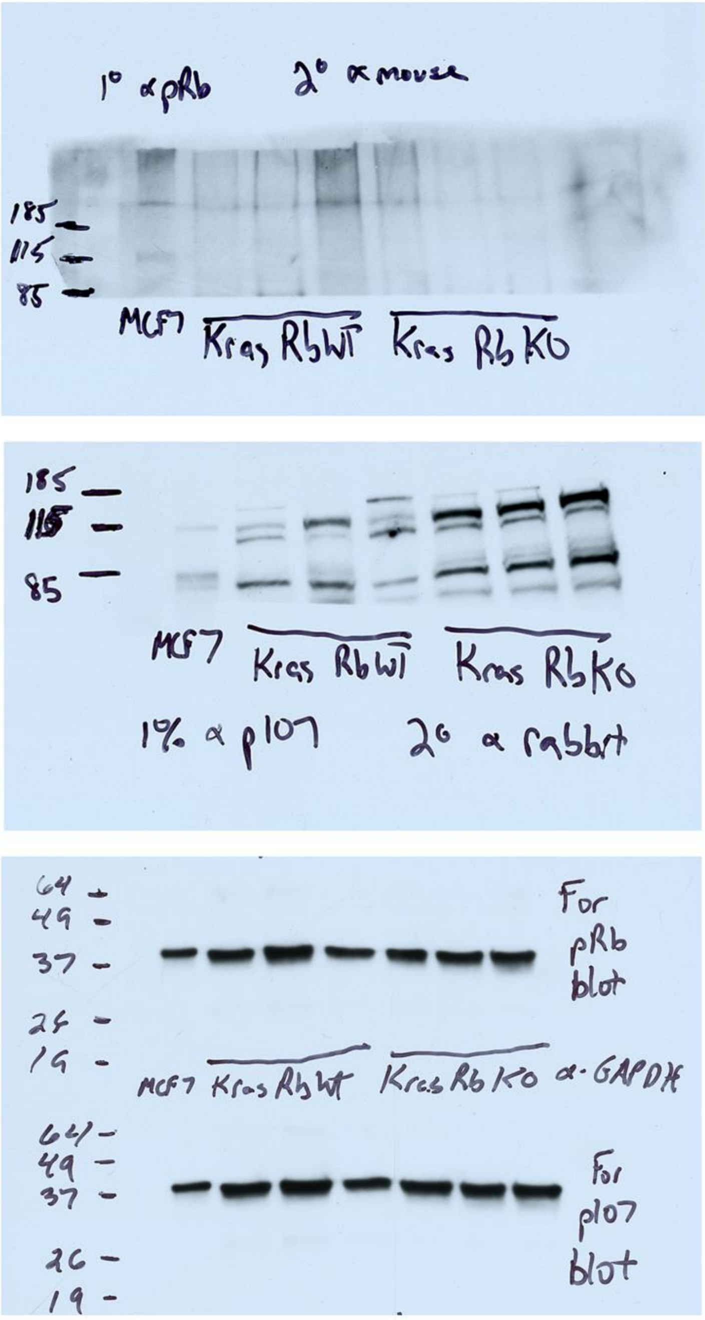

Full scans of all Western blots. Membranes were cut between the 85 and 64kDa range within the molecular weight markers for simultaneous assessment of Rb1 (~110kDa), p107 (~115kDa), and GAPDH (~37kDa). Non-specific bands were detected at ~200kDa and 85kDa within the Rb1 or p107 immunoblots, respectively.

Reference

1. Ho, V.M.; Schaffer, B.E.; Karnezis, A.N.; Park, K.S.; Sage, J. The retinoblastoma gene Rb and its family member p130 suppress lung adenocarcinoma induced by oncogenic K-Ras. *Oncogene* **2009**, *28*, 1393–1399.

© 2020 by the authors. Licensee MDPI, Basel, Switzerland. This article is an open access article distributed under the terms and conditions of the Creative Commons Attribution (CC BY) license (http://creativecommons.org/licenses/by/4.0/).
